# Carbon nanotube-wrapped Fe_2_O_3_ anode with improved performance for lithium-ion batteries

**DOI:** 10.3762/bjnano.8.69

**Published:** 2017-03-17

**Authors:** Guoliang Gao, Yan Jin, Qun Zeng, Deyu Wang, Cai Shen

**Affiliations:** 1 Ningbo Institute of Materials Technology & Engineering Chinese Academy of Sciences. 1219 Zhongguan Road, Zhenhai District, Ningbo, Zhejiang, China; 2 Guangzhou Key Laboratory for Special Fiber Photonic Devices, South China Normal University, Guangzhou 510006, China; 3 Laboratory of Nanophotonic Functional Materials and Devices, School of Information and Photoelectronic Science and Engineering, South China Normal University, Guangzhou 510006, China

**Keywords:** anode material, carbon nanotubes, hydrothermal synthesis method, lithium-ion batteries

## Abstract

Metall oxides have been proven to be potential candidates for the anode material of lithium-ion batteries (LIBs) because they offer high theoretical capacities, and are environmentally friendly and widely available. However, the low electronic conductivity and severe irreversible lithium storage have hindered a practical application. Herein, we employed ethanolamine as precursor to prepare Fe_2_O_3_/COOH-MWCNT composites through a simple hydrothermal synthesis. When these composites were used as electrode material in lithium-ion batteries, a reversible capacity of 711.2 mAh·g^−1^ at a current density of 500 mA·g^−1^ after 400 cycles was obtained. The result indicated that Fe_2_O_3_/COOH-MWCNT composite is a potential anode material for lithium-ion batteries.

## Introduction

The depletion of non-renewable energy resources such as coal, petrol and natural gas has led to the urgent need to use sustainable and renewable energies. In comparison to dry cells, lithium-ion batteries (LIBs) have the unique advantage of low working voltage, long cycle life and high energy density. LIBs have found wide application as power storage solution for portable electronic devices, hybrid electric vehicles and battery electric vehicles [1–8]. Graphite, is the most commonly used anode material for LIBs, has a theoretical specific capacity of 372 mAh·g^−1^ [9], which does not meet the requirements of hybrid electric vehicles. Thus, the development of next-generation batteries with low production cost, high energy density, high safety standards and good performance is of great interest.

Fe_2_O_3_ is one of the most promising materials for the use as anode materials in LIBs, because it offers a high theoretical capacity (1005 mAh·g^−1^) [10], is widely available inexpensive and environmental friendly, and exhibits an excellent redox activity [11–18]. The redox reaction of Fe_2_O_3_ with lithium is as follows:


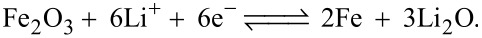


Various methods have been reported for the synthesis of Fe_2_O_3_. Cho et al. [19] reported the synthesis of α-Fe_2_O_3_ materials by a simple high-temperature processing. of composite materials. In this method, an Fe-based metal organic framework [MIL-88B (Fe)] was used as a precursor for the synthesis of spindle-like α-Fe_2_O_3_ nanoparticles with a mesoporous structure of less than 20 nm. When used as anode material for LIBs, these nanoparticles demonstrated a capacity of 911 mAh·g^−1^ at a current density of 200 mA·g^−1^ even after 50 cycles. As the current density was increased to 10 A·g^−1^, a discharge capacity of 424 mAh·g^−1^ was obtained. However, the capacity of this material declined gradually with increased number of cycles due to the low conductivity of Fe_2_O_3_. Liang et al. [20] employed a simple and easy hydrothermal method for the synthesis of α-Fe_2_O_3_ microspheres by using sodium citrate as surfactant. A reversible discharge capacity of 489.5 mAh·g^−1^ was obtained at a current density of 100 mA·g^−1^ of up to 50 cycles. The specific capacity of the synthesized α-Fe_2_O_3_ microspheres was much better than that of commercial graphite (372 mAh·g^−1^) although further improvement in cyclic stability was needed [20].

An effective method to improve the electrical conductivity of Fe_2_O_3_ is to fabricate Fe_2_O_3_/carbon nanotube (CNT), Fe_2_O_3_/graphene or Fe_2_O_3_/graphene/CNT composites, which have been demonstrated to have improved cycling performance [21–25]. All of these materials demonstrated considerable specific capacity, but with some drawbacks such as complex synthesis methods [21–22], high cost and low cycle life. So it is essential to find new facile preparation methods. Yu et al. [26] successfully embedded Fe_2_O_3_ nanoparticles inside CNTs, which reduced the volume change of Fe_2_O_3_ nanoparticles during charge/discharge. A highly reversible conversion reaction between Fe^0^ and Fe^3+^ (Fe_2_O_3_) during lithiation/delithiation can also be observed. The synthesized Fe_2_O_3_/CNT has successfully overcome the shortcoming of low electrical conductivity of Fe_2_O_3_. Zhou et al. [27] has fabricated Fe_2_O_3_@GS by using a simple spray drying method, which significantly improved the capacity of Fe_2_O_3_ albeit with a low cycling performance. Ye et al. [28] applied a solvent-directed sol–gel method to prepare graphene-wrapped Fe_2_O_3_, which demonstrated an excellent capacity retention of 777 mAh·g^−1^ at a current density of 100 mA·g^−1^ after 30 cycles. Wang et al. [29] synthesized Fe_2_O_3_/GCNTs via a hydrothermal synthesis method and subsequent thermal reduction. The synthesized Fe_2_O_3_/GCNTs exhibited a high reversible capacity of 716 mAh·g^−1^ at 50 mA·g^−1^ after 120 cycles. Nevertheless, these materials demonstrated low capacity at high current densities.

In this paper, we have used ethanolamine as precursor to prepare Fe_2_O_3_/COOH-MWCNT composites through a simple hydrothermal synthesis. Hydrothermal syntheses are frequently used to obtain composite oxides with uniform particle size distribution. The synthesized material can effectively buffer volume change caused by charge and discharge; and improve the electrical conductivity of the electrode [26,29–34].

## Experimental

### Synthesis of metal hydroxide composite

The composite was synthesized via a simple hydrothermal method. Firstly, iron(III) chloride hexahydrate (FeCl_3_·6H_2_O, 2.1624 g, ≥99.0%, Sinopharm Chemical Reagent Co, Ltd) and ethanolamine were each dissolved separately 100 mL distilled water and stirred for 30 min to obtain uniform solutions. Following that, the iron(III) chloride solution was added dropwise into the ethanolamine solution. The solution was then kept at room temperature for three days to obtain orange metal hydroxide solution.

### Synthesis of Fe_2_O_3_-CMWCNT composites

**Scheme 1:**
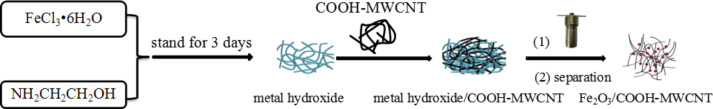
Preparation of Fe_2_O_3_/COOH-MWCNT composites.

Approximately 50 mL of the aforementioned solution was added to 16.67 mL aqueous solution of 2 mg/mL short carboxyl of multi-walled carbon nanotubes (COOH-MWCNT Aladdin Corp). The mixture was magnetically stirred for 12 h. Finally, the mixture was transferred into teflon-lined stainless steel autoclave, and kept at 150 °C for 24 h. The autoclave was then cooled to room temperature. The obtained precipitates were filtered, washed with distilled water for several times and kept in 30 mL of DMF solution to remove ethanolamine. Finally, the obtained black solid powder was washed with ethanol solution for several times and fully dried in a vacuum oven at 80 °C for 10 h. Details of the synthesis process of the Fe_2_O_3_-CMWCNT are presented in Scheme 1.

### Morphological and structural characterization

The as-prepared Fe_2_O_3_/COOH-MWCNT were characterized using X-ray diffractometry (XRD, D8 Discover, Broker AXS, Cu radiation, λ = l.540596 Å), scanning electron microscopy (SEM, S-4800, Hitachi), transmission electron microscopy (TEM, Tecnai F20) and thermogravimetric analysis (TGA, Perkin Elmer TGA 7).

### Electrochemical measurements

Working electrodes were fabricated by a standard slurry casting procedure. The as-prepared anode material (70 wt %), conductive carbon black (Super P, 20 wt %) and polyvinylidene fluoride (PVDF, 10 wt %) were mixed in *N*-methyl-2-pyrrolidone to form a uniform slurry that was and stirred for 6 h. The slurry was spread on to the surface of a copper foil using a medical blade. The casted film was heated at 110 °C for 12 h in a vacuum oven to evaporate the residual solvent. After cooling down to room temperature, the casted film was cut into small round shape (average diameter of 14 mm) and used as anode. Metallic lithium was used as counter electrode as well as reference electrode. The cells separator was Celgard 2400 polypropylene film. Electrolyte was prepared by dissolving 1 M lithium hexafluorophosphate (LiPF_6_) in a mixed solution of fluoroethylene carbonate/ethyl methyl carbonate/dimethyl carbonate (FEC/EMC/DMC, 1:1:1 by volume). Coin cells (2032 type) were assembled inside an argon filled glove box with a moisture and oxygen levels of less than 0.1 ppm. The assembled cells were kept at room temperature for 12 h before electrochemical performance test

Electrochemical performance of the assembled cells were then tested by galvanostatic charge/discharge measurements, cyclic voltammetry (CV) and electrochemical impedance spectroscopy (EIS). Charge/discharge tests of the assembled cells were carried out using a commercial battery test system (LAND model, CT2001A) at a constant current in the potential range of 0.01–3.00 V (vs Li/Li^+^). The cyclic voltammetry measurements were conducted in the same potential window at a scanning rate of 0.05 mV·s^−1^. Electrochemical impedance spectroscopy was carried out at a frequency range 10 mHz to 1 MHz with an AC amplitude of 10 mV. Both CV and EIS measurements were carried out on an electrochemical workstation (Ametek 1470E)

## Results and Discussion

**Figure 1:**
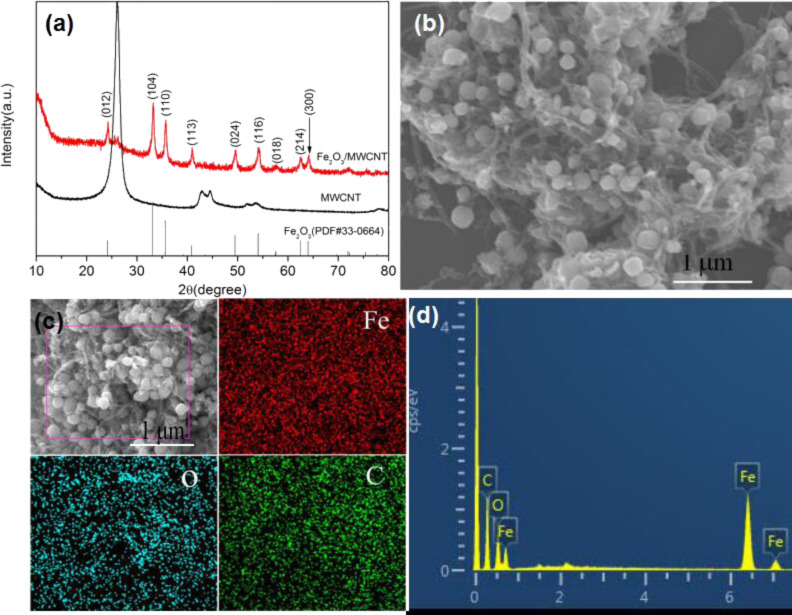
Fe_2_O_3_/COOH-MWCNT composites: (a) XRD patterns; (b) SEM image; (c) and (d) energy-dispersive X-ray analysis (EDX).

Figure 1a shows XRD spectra of Fe_2_O_3_/COOH-MWCNT composites and COOH-MWCNT. All peaks of Fe_2_O_3_ can be assigned to rhombohedral α-Fe_2_O_3_ (JCPDS No. 33-0664), indicating the well-crystalline structure of the as-prepared Fe_2_O_3_ nanoparticles. The black spectrum refers to carbon nanotubes, and the peak at 26° is the characteristic peak of carbon nanotubes. No obvious impurity peak was observed, indicating that high crystallinity and purity of the synthesized materials. Figure 1b shows SEM images of the as-prepared Fe_2_O_3_ composites, which consist of Fe_2_O_3_ nanoparticles (ca. 100–200 nm) evenly distributed within the MWCNT networks. Such networks are advantageous for ionic migration. Figure 1c,1d shows the energy dispersive X-ray analysis (EDX) of the Fe_2_O_3_/MWCNT composite. Three distinct elements (C, O and Fe) can be observed. All the elements were uniformly distributed. The aforementioned results demonstrated that Fe_2_O_3_ nanoparticles were evenly distributed over the COOH-MWCNT frameworks.

**Figure 2:**
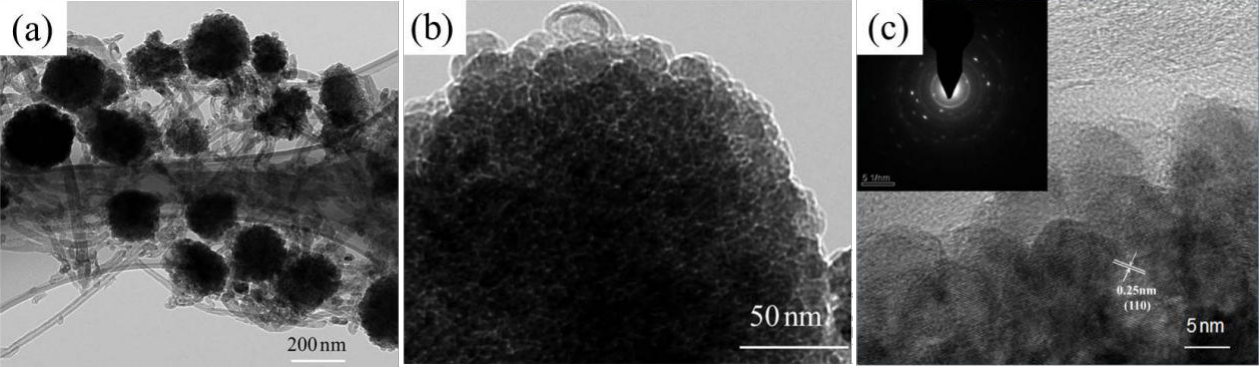
Fe_2_O_3_/COOH-MWCNT composites: (a) and (b) HRTEM; (c) HRTEM and selected area electron diffraction patterns of TEM.

The structure of the as-prepared Fe_2_O_3_/COOH-MWCNT composite material was further examined by TEM and HRTEM. The results are presented in Figure 2. It can be seen in Figure 2a that Fe_2_O_3_ particles with a size of around 200 nm are evenly distributed between COOH-MWCNTs, which is in good agreement with the SEM result. Figure 2b shows a HRTEM image of the composites. There is an obvious secondary structure in which smaller Fe_2_O_3_ nanoparticles are formed on larger Fe_2_O_3_ nanoparticles. Figure 2c shows a HRTEM image and a selected area electron diffraction pattern of the Fe_2_O_3_ nanoparticles. Smaller secondary particles are clearly observed. The measured (110) lattice spacing is 0.25 nm, which is in agreement with [35].

**Figure 3:**
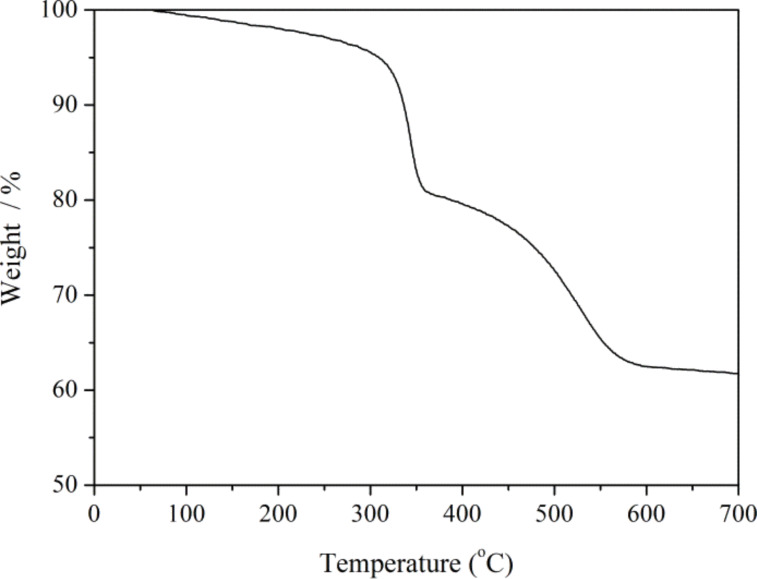
TG curve of Fe_2_O_3_/COOH-MWCNT composites.

The thermal stability of the as-prepared Fe_2_O_3_/COOH-MWCNT composites was measured using a thermogravimetric analyzer. Thermogravimetric analysis (TGA) was carried out in oxygen by heating the samples to 700 °C at a heating rate of 10 °C·min^−1^ (Figure 3). A weight loss of approximately 6% can be observed at a temperature of 100 °C indicating a loss of absorbed water in the sample. Fe_2_O_3_/COOH-MWCNT composites decomposed in two steps in a temperature range of 300–570 °C. The decomposition of the organic part can be observed in a temperature range of 300 to 360 °C, and resulted in 13% weight loss [10]. The oxidative breakdown of COOH-MWCNTs can be observed in a temperature range of 360–570 °C. The mass of the as-prepared material did not change at temperatures above 600 °C.

**Figure 4:**
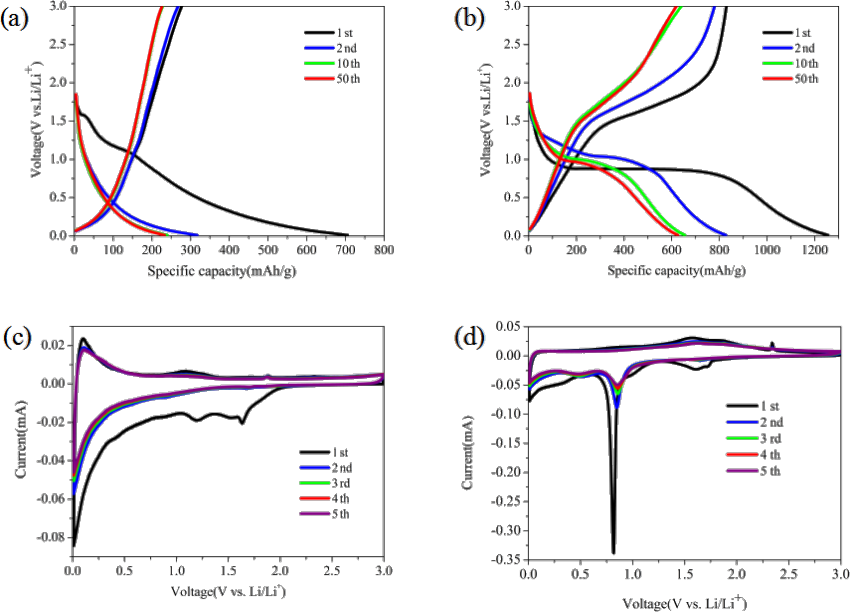
Electrochemical performance of the electrodes: (a) Discharge–charge profiles of COOH-MWCNT at a current density of 500 mA·g^−1^; (b) discharge–charge profiles of Fe_2_O_3_/COOH-MWCNT composites at a current density of 500 mA·g^−1^; (c) cyclic voltammograms of COOH-MWCNT at a scan speed of 0.05 mV·s^−1^; (d) cyclic voltammograms of Fe_2_O_3_/COOH-MWCNT at a scan speed of 0.05 mV·s^−1^.

Figure 4 depicts the electrochemical performance of COOH-MWCNT composites (Figure 4a,c) and Fe_2_O_3_/COOH-MWCNT (Figure 4b,d). Figure 4a reveals the discharge–charge profiles of COOH-MWCNT at 500 mA·g^−1^. In the first discharge curve from open-circuit voltage to 0.01 V, Li^+^ ions were inserted into the electrode material. As the reaction proceeded, two weak discharge plateaus were observed at about 1.2 V and 1.6 V, which were attributed to the reactions between functional groups of carbon nanotubes and lithium metal. The initial discharge and charge capacities of COOH-MWCNT were 710 and 300 mAh·g^−1^ with a coulombic efficiency of 42%. The large capacity fading and low coulombic efficiency observed for the electrode in the first cycle can be ascribed to irreversible processes such as formation of a solid–electrolyte interface (SEI) film and the decomposition of electrolyte [9–10]. The 10th and 50th discharge curves almost coincide with the 2nd discharge curve, which can be attributed to the high conductivity of carbon nanotubes.

Figure 4b shows charge and discharge profiles of the Fe_2_O_3_/COOH-MWCNT composites. The first discharge curve of cells exhibited an apparent plateau at around 0.8 V. During recharging, there was a reversible oxidation from Fe^0^ to Fe^3+^ with a plateau at 1.5–1.9 V. Both plateaus are typical features for Fe_2_O_3_ materials (Fe_2_O_3_ + 6Li^+^ + 6e^−^ 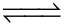 2Fe + 3Li_2_O) [26,29,36]. The first charge and discharge capacities of the composite material were 850 mAh·g^−1^ and 1250 mAh·g^−1^, respectively, with a coulombic efficiency of 68%. A large irreversible capacity loss of 400 mAh·g^−1^ might be related to formation of SEI and other side reactions. In the second cycle, the potential plateaus were detected at 1.5–1.7 and 0.9–1.1 V, which are consistent with previously reported results [29]. The coulombic efficiency increased to 95% in the second cycle, which continued to increase steadily in subsequence cycling indicating a good stability of the composites.

In order to measure electrochemical activity and oxidation/reduction potential, cyclic voltammograms of Fe_2_O_3_/COOH-MWCNT composites was measured and the results were displayed in Figure 4c,d. Figure 4c shows the cyclic voltammogram of COOH-MWCNT at a scanning rate of 0.05 mV·s^−1^. In the first charge and discharge process, two anodic peaks at about 1.7 V and 1.2 V were observed, which is consistent with the charge and discharge curves observed in Figure 4a. In the first cycle CV of Fe_2_O_3_/COOH-MWCNT composites (Figure 4d), there is a sharp peak at about 0.8 V, which can be ascribed to the formation of a SEI as well as reduction of Fe^3+^ into Fe^0^ [15,17–18,26,32,37]. The peaks at around 0.9 V are lithium storage peaks. The high intensity reductive peak at around 1.7 V during the first cycle became very weak during subsequent cycles indicating that an irreversible reaction only happened during the first cycle. This might be due to the reaction between functional groups of carbon nanotubes and lithium metal. Moreover, the composite electrode shows anodic peaks at about 1.5–1.9 V, which can be attributed to the oxidation of Fe (Fe^0^ to Fe^3+^). The peak at about 0.8 V also shifted to a more positive potential of 0.86 V due to polarization [19,29]. After the first CV, all the subsequent CV curves coincide with the second curve indicating good electrochemical stability.

**Figure 5:**
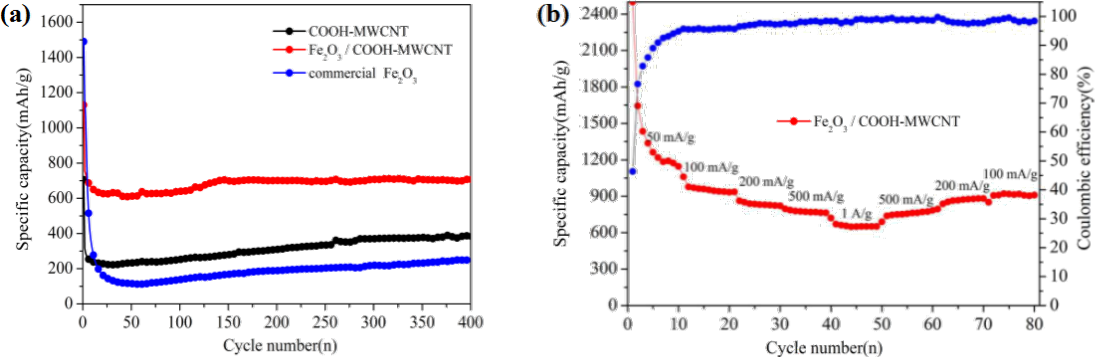
Electrochemical performance of the Fe_2_O_3_ electrode: (a) Comparisons of the cycling performance among COOH-MWCNT, Fe_2_O_3_/COOH-MWCNT composites and commercial Fe_2_O_3_ at a current density of 500 mA·g^−1^; (b) rate capability of Fe_2_O_3_/COOH-MWCNT composites.

Figure 5a shows comparisons of the cycling performance among COOH-MWCNT, Fe_2_O_3_/COOH-MWCNT composite and commercial Fe_2_O_3_ at a current density of 500 mA·g^−1^. A rising trend after an initial capacity drop can be observed for the Fe_2_O_3_/COOH-MWCNT composite. This is a common phenomenon in transition metal oxide anode materials. It can be ascribed to the penetration of the electrolyte and gradual exposure of the active sites [38–40]. The discharge capacity of the Fe_2_O_3_/COOH-MWCNT composite stabilized at 711.2 mAh·g^−1^ at a current of 500 mA·g^−1^ following 400 cycles, which is superior to that of COOH-MWCNT and commercial Fe_2_O_3_. The Fe_2_O_3_/COOH-MWCNT composite showed excellent cycle stability due to the high surface area and flexibility of carbon nanotubes, which is capable of reducing the volume change of metal oxide during charging and discharging [41]. In comparison to work done by Chen et al. [41], our synthesis method does not include sintering and is therefore less energy consuming. Liu et al. [24] and Zhou et al. [27] reported the synthesis of Fe_2_O_3_ followed by addition into GO suspension, which resulted in uneven dispersion of Fe_2_O_3_ in the GO suspension and reduced cycling performance. Different from the previous works by Liu et al. [24] and Zhou et al. [27], the metal hydroxides in the present work were synthesized in aqueous solution followed by mixing with carbon nanotubes, which ensured an even distribution of Fe_2_O_3_ particles onto the carbon nanotubes.

Figure 5b shows the rate performance test of the as-prepared Fe_2_O_3_/COOH-MWCNT composite. A highly symmetric pattern was obtained (except for initial several cycles due to the complicated side reactions and irreversible reactions). As the current density increased to 1000 mA·g^−1^, the discharge capacity can reach 650 mAh·g^−1^. When the current density dropped to 100 mA·g^−1^, the capacity returned to 920 mAh·g^−1^, with a coulombic efficiency as high as 99%, showing a good high-rate discharge ability and cycle stability. It can be inferred that COOH-MWCNTs play an important role to maintain structural integrity and to improve the electrical conductivity of the hybrid structure. In comparison to Yu et al. [23] and Yu et al. [26], the hydrothermal method used in our work is easy-to-handle and can be easily scaled up. Secondly, our capacity was also higher than previously reported values. The discharge capacity was stabilized at 711.2 mAh·g^−1^ at a current of 500 mA·g^−1^ after 400 cycles. Meanwhile, Yu et al. [26] reported a discharge capacity of less than 600 mAh·g^−1^ at a current of 500 mA·g^−1^. The good electrochemical cycle performance is probably due to the high capacity of Fe_2_O_3_ and good conductivity of COOH-MWCNTs.

**Figure 6:**
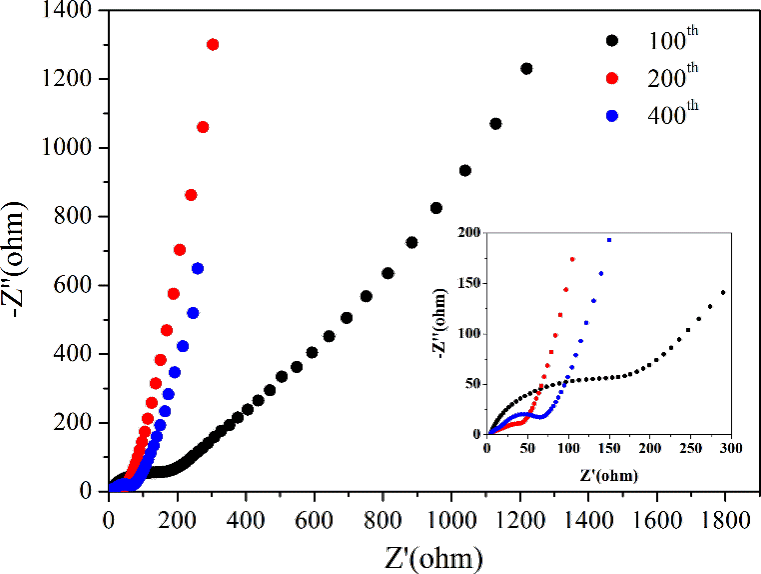
Nyquist plots of Fe_2_O_3_/COOH-MWCNT composite electrodes after 100, 200 and 400 discharge–charge cycles at a current density of 500 mA·g^−1^.

EIS experiments were carried out to estimate the enhanced Li^+^ storage performance of the composites in the frequency region from 100 MHz to 0.01 Hz at room temperature. As shown in Figure 6, the Nyquist plots show a depressed semicircle at high frequency and a straight line at low frequency. The diameter of the depressed semicircle represents the resistance of the charge-transfer process, while the straight line can be assigned to the diffusion of lithium ion in the electrode [29]. It can be found that the resistance of the charge transfer process after the 200th cycle was much smaller than that after the 100th cycle for Fe_2_O_3_/COOH-MWCNT composites, indicating a good charge transfer in the electrode. The resistance after the 400th cycle demonstrated a slight increase, indicating that the composites can keep a high capacity and a good cycle stability.

## Conclusion

In summary, we have successfully fabricated an Fe_2_O_3_/COOH-MWCNT composite material using ethanolamine and iron chloride hexahydrate as precursors. Fe_2_O_3_ nanoparticles with secondary structures were evenly distributed in the COOH-MWCNT network. A reversible capacity of around 711.2 mAh·g^−1^ was maintained at a current density of 500 mA·g^−1^ after 400 cycles, which is much higher than that of COOH-MWCNT composite and commercial Fe_2_O_3_. The result indicated that Fe_2_O_3_/COOH-MWCNT composite can be a potential anode material for lithium-ion batteries.
